# How Does Fearful Emotion Affect Visual Attention?

**DOI:** 10.3389/fpsyg.2020.584412

**Published:** 2021-01-08

**Authors:** Zhe Shang, Yingying Wang, Taiyong Bi

**Affiliations:** ^1^Department of Human Resource Management, School of Government, Beijing Normal University, Beijing, China; ^2^Academy for Advanced Interdisciplinary Studies, Peking University, Beijing, China; ^3^School of Management, Zunyi Medical University, Zunyi, China

**Keywords:** emotion, attention, visual acuity, fearful emotion, perceptual facilitation

## Abstract

It has long been suggested that emotion, especially threatening emotion, facilitates early visual perception to promote adaptive responses to potential threats in the environment. Here, we tested whether and how fearful emotion affects the basic visual ability of visual acuity. An adapted Posner’s spatial cueing task was employed, with fearful and neutral faces as cues and a Vernier discrimination task as the probe. The time course of the emotional attention effect was examined by varying the stimulus onset asynchrony (SOA) of the cue and probe. Two independent experiments (Experiments 1 and 3) consistently demonstrated that the brief presentation of a fearful face increased visual acuity at its location. The facilitation of perceptual sensitivity was detected at an SOA around 300 ms when the face cues were presented for both 250 ms (Experiment 1) and 150 ms (Experiment 3). This effect cannot be explained by physical differences between the fearful and neutral faces because no improvement was found when the faces were presented inverted (Experiment 2). In the last experiment (Experiment 4), the face cues were flashed very briefly (17 ms), and we did not find any improvement induced by the fearful face. Overall, we provide evidence that emotion interacts with attention to affect basic visual functions.

## Introduction

Possibly due to its critical role in evolution and individual survival (Ohman and Mineka, 2001), emotional information is processed in priority to a large extent. Evidence has shown that emotional faces get into consciousness faster and more efficiently than neutral faces when presented subconsciously (Alpers and Gerdes, 2007; Bannerman et al., 2008; Amting et al., 2010). Threatening targets including fearful and angry faces, spiders, and snakes are detected faster and affected less by the existence of distractors (Hansen and Hansen, 1988; Mogg and Bradley, 1999; Fox et al., 2000; Mogg et al., 2000; Eastwood et al., 2001; Ohman et al., 2001). Besides, emotional stimuli captured attention so easily that it could escape the attentional blink effect (Anderson and Phelps, 2001). In accordance, neural evidence has demonstrated exceptionally fast processing of threatening information in humans’ and primates’ brains (Morris et al., 1999; Tamietto et al., 2012; Zhang et al., 2013; Mendez-Bertolo et al., 2016), confirming the high priority of emotion processing.

Emotion’s particularity is not only working on the perceptual facilitations on its own, but also by its influence on other processes. One most widely acknowledged effect is emotion’s modulation on perceptual processing (Bayer et al., 2018; Quek et al., 2018). The first evidence was from Phelps et al. (2006) who presented fearful or neutral face cues before Gabor patches presented at low contrast and measured participants’ contrast sensitivity change due to emotional attention. Results showed an increased contrast sensitivity of Gabor patches following the fearful face cues. Importantly, this effect was modulated by the amount of attention attributed to the location. Inspired by this study, Bocanegra and Zeelenberg (2009) explored the effect of emotional attention on another low-level visual property, orientation sensitivity. However, in their results, fearful face cues triggered not only facilitation but also interference on orientation sensitivity of the target Gabor patch. The effect varied as a function of the spatial frequency of the Gabor patch. They explained this two-way effect as inhibitory interactions between magnocellular and parvocellular pathways which encode low and high spatial-frequency information respectively. More recently, another study (Lee et al., 2014) systematically examined the effect of emotional arousal on individuals’ contrast sensitivity function and demonstrated a shift to lower spatial frequencies under high emotional arousal. So far, threatening stimuli have been found to modulate visual sensitivity (Phelps et al., 2006; Bocanegra and Zeelenberg, 2009), increase temporal resolution at the expense of fine-grained spatial vision (Bocanegra and Zeelenberg, 2011b), and elongate time perception (Yamada and Kawabe, 2011). These findings all support the seemingly surprising effect of emotion on low-level perceptual sensitivity. More than that, such effects have also been indicated by the neural evidence. Neuroimaging studies have shown that emotional stimuli enhanced neural activity in the primary visual cortex (Padmala and Pessoa, 2008). Emotional stimuli have also been shown to enhance as early as 60 to 90 ms components in several event-related potential studies (Rossi and Pourtois, 2012).

The priority of emotion, as well as its influence on perceptual processing, is mainly/mostly due to its interactions with attention (Vuilleumier and Huang, 2009). Researchers have suggested that people more readily pay attention to emotional than neutral stimuli (Vuilleumier et al., 2001; Fox et al., 2002). This is supported by neural evidence that the emotion system interacts with the attentional system to affect perceptual processing. For example, when fearful faces enhanced neural activity in face-processing regions, attention was found to increase this enhancement (Vuilleumier et al., 2001; Pessoa et al., 2002; Anderson et al., 2003). Neuroscience studies have also shown that attention and emotion both amplify the sensory processing of the target items in a similar manner (Pourtois et al., 2013).

However, the interactions between emotion and attention is complicated. For example, while threatening cues are suggested to facilitate perceptual detection of targets at the same location (Fox et al., 2001), researchers have occasionally failed to detect such attentional benefits effect but instead observed interference effect in threatening faces. The interference effect might be due to emotion processing *per se* could occupy the attentional resources. Koster et al. (2007) found delayed responding on trials with briefly presented emotional cues, suggesting stronger allocation of cognitive resources to briefly-presented emotional faces and lower level of attentional processing at other non-emotional locations. In accordance, it has been reported that for people with anxiety disorders, who are more susceptible to negative emotions, attentional processes take a longer time to disengage from threat-related stimuli and thus were processed slower (Fox et al., 2001). Additionally, trait anxiety mediates the effect that emotion potentiates the effects of exogenous attention, with stronger influences of attention and emotion in anxious observers (Barbot and Carrasco, 2018). This delayed attention disengagement has also been revealed in normal people (Nummenmaa et al., 2006) and during natural viewing (Calvo and Lang, 2004; Acunzo and Henderson, 2011).

Therefore, when emotion processing takes up the attentional resources, emotional attention might instead interfere with other processes. The effect of emotional attention might vary in time. The temporal dynamics of attentional effect has been widely studied for traditional attentional manipulations (Posner and Cohen, 1984; Huang et al., 2015). Notably, the emotion-attention interactions on basic perception received much attention recently (Bocanegra and Zeelenberg, 2011a; Ferneyhough et al., 2013; Barbot and Carrasco, 2018). Several researchers examined the time course of the emotional effect in normal and psychopathological populations. A study among individuals with high trait anxiety examined the time-course of the overall attention effect in the dot-probe task, and found greater attentional effect for threatening words at time-points ranging from 100 to 1500 ms (Mogg et al., 1997). Similarly, several studies have found evidence of persisting attentional vigilance to threat in high-anxious individuals at SOAs between 100 and 1500 ms (Bradley et al., 1998; Derryberry and Reed, 2002). For example, Derryberry and Reed (2002) found the effect at an SOA of 250 ms among trait-anxiety individuals, which were reduced at 500 ms. Evidence showed an early attentional bias (≤ 500 ms SOA) to threatening images among high trait anxiety individuals (Koster et al., 2005; Mogg and Bradley, 2006). Studies on healthy populations showed similar results. Cooper and Langton (2006) examined the effects at durations of 100 and 500 ms, indicating that selective attention to threat may be faster in controls than in psychopathological individuals. Torrence et al. (2017) used a three dot-probe task and found that fearful and happy faces captured and held attention. Three different SOAs (133, 266, and 532 ms) and four different SOAs (84, 168, 336, and 672 ms) were adopted in two experiments with a brief presentation of the face cue (133 ms). Results indicated that happy faces captured and held attention on a time-course (SOA) from 168 to 336 ms. While fearful faces also captured and held the attention, but at a slightly different SOAs from 84 to 266 ms, indicating that attention is captured and held at times earlier than approximately 300 ms after the presentation of fearful faces. To sum up, these findings on the attentional processing of threat signals reveal attentional effects between 100 and 500 ms post-stimulus onset (Koster et al., 2005; Cooper and Langton, 2006; Mogg and Bradley, 2006).

The current study aimed to understand the influence of emotional attention on perceptual processing. Specifically, we tested whether threatening emotion also affects the low-level visual ability of visual acuity. An adapted Posner’s spatial cueing paradigm (Posner, 1980) was employed with fearful and neutral faces being used as cues and a Vernier discrimination task being used as the probe. Fearful faces were used to manipulate emotional attention by the consideration that they indicate a vague threat in the environment (Phelps et al., 2006). The Vernier discrimination task, which was used to measure visual acuity, was given either at the same or opposite position of the fearful face cue. In this way, the effect of emotion on visual acuity could be measured. Two additional manipulations were carried out to understand the interaction between emotion and attention. First, we varied the interval between the face cues and the visual acuity probe within each experiment so that we could examine the time course of the emotional attention effect. Second, we manipulated the duration of the face cues such that the fearful face could either be fully processed or not. While the previous studies suggested that people see better when being primed by emotional stimuli (Phelps et al., 2006; Lee et al., 2014), we hypothesized that it depends. In summary, we hypothesized that: (1) threatening faces may facilitate perceptual discrimination at an early stage of emotional face processing; (2) the cueing effect may be largely disrupted by turning faces upside down; (3) when the fearful face is presented very briefly (17 ms), the threatening information may not facilitate perceptual discrimination, and may instead cost attention and even damage visual acuity.

## Experiment 1

A modified Posner spatial cueing paradigm was used (Posner, 1980) with fearful and neutral faces as cues and a Vernier task as the probe. While the time course of the attentional effect has been widely studied (Posner and Cohen, 1984; Huang et al., 2015), it remains unknown about the temporal dynamics of the emotional attention effect. Here, we also manipulated the stimulus onset asynchrony (SOA) and tested the effect at seven SOAs.

### Methods

#### Participants

Twenty naïve participants (11 females) with normal or corrected to normal vision were recruited from Peking University. All participants were right-handed and had no known neurological or visual disorders. They gave written, informed consent in accordance with procedures and protocols approved by the human subject review committee of Peking University.

#### Apparatus and Stimuli

Stimuli were presented on an IIYAMA color graphic monitor (model: MM906UT; refresh rate: 60 Hz; resolution: 1024 ^∗^ 768; size: 19 in.) (Zhang et al., 2018). Participants viewed the stimuli from a distance of 57 cm with their head stabilized on a chin rest. Face images were derived from the database of Chinese Facial Affective Picture System (CAFPS) (Gong et al., 2011). This database consists of 600 gray-scaled face images, with 200 for positive, negative and neutral expression respectively. We used the 200 negative and 200 neutral images (100 images for each gender) for stimuli selection. Forty images (20 fearful and 20 neutral), which were selected for each participant individually, were used in the main experiment. The stimuli were equalized in luminance, rooted-mean-square contrast, and size. All the stimuli were presented on a gray background and extended 4.8 deg ^∗^ 5.6 deg.

#### Procedure and Design

Stimuli selection was performed before the main experiment, in which all the 400 images were presented for participants to rate how fearful each face was on a 7-point Likert scale. Twenty most fearful (10 males and 10 females) and twenty most peaceful faces (10 males and 10 females) were then selected for each participant individually.

The main experiment began immediately after the stimuli selection. Each trial started with the presentation of a fixation cross at the center of the screen for 500∼1500 ms against a black background. Then two face images were presented to the left and right sides of the fixation cross with an eccentricity of 7 degrees. The face images disappeared after 250 ms, followed by a blank screen. Seven durations (i.e., 17, 50, 100, 150, 250, 500, and 1000 ms) were used for the blank screen in order to investigate the time course of the emotional attention effect. Accordingly, the stimulus onset asynchrony (SOA) varied among 267, 300, 350, 400, 500, 750, and 1250 ms. To test the emotional attention effect on visual acuity, a Vernier discrimination task was given immediately afterward. In each trial, two dots were presented on one side of the visual field for 67 ms. The upper dot was misaligned 0.07 deg leftward or rightward as to the lower dot, and the center of the dots coincided with the center of the preceding face. Subjects were asked to judge whether the upper dot was to the left or right side of the lower dot.

**FIGURE 1:**
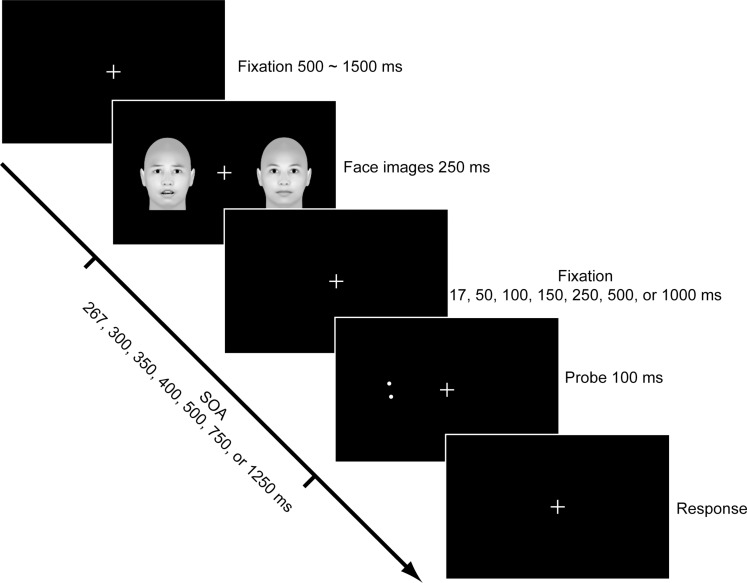
The Experimental procedure.

A modified Posner cueing paradigm was used to assess the emotional attention effect induced by the fearful faces. We used a 3 (cue types: valid, invalid, and neutral) × 7 (SOAs) within-subject design. The side where a fearful face was presented served as the emotional attention cue. Thus, for the valid cue condition, the Vernier probe was presented on the same side of the fearful face; for the invalid cue condition, the Vernier probe was presented at the opposite side of the fearful face; while for the neutral cue condition, both of the faces were neutral and thus had no emotional cue. The experimental procedure is shown in Figure 1. Trials for different conditions were presented in a random order, and both the fearful faces and the Vernier probe were presented at the left and right side with a chance of 50%. The fearful and neutral faces for each trial were randomly selected from the 40 images chosen at the beginning of the experiment. Each participant completed 80 trials for each condition (1680 trials in total). Accuracy and response time (RT) were collected for each subject except the RT for one subject due to technical reason.

### Results

**FIGURE 2:**
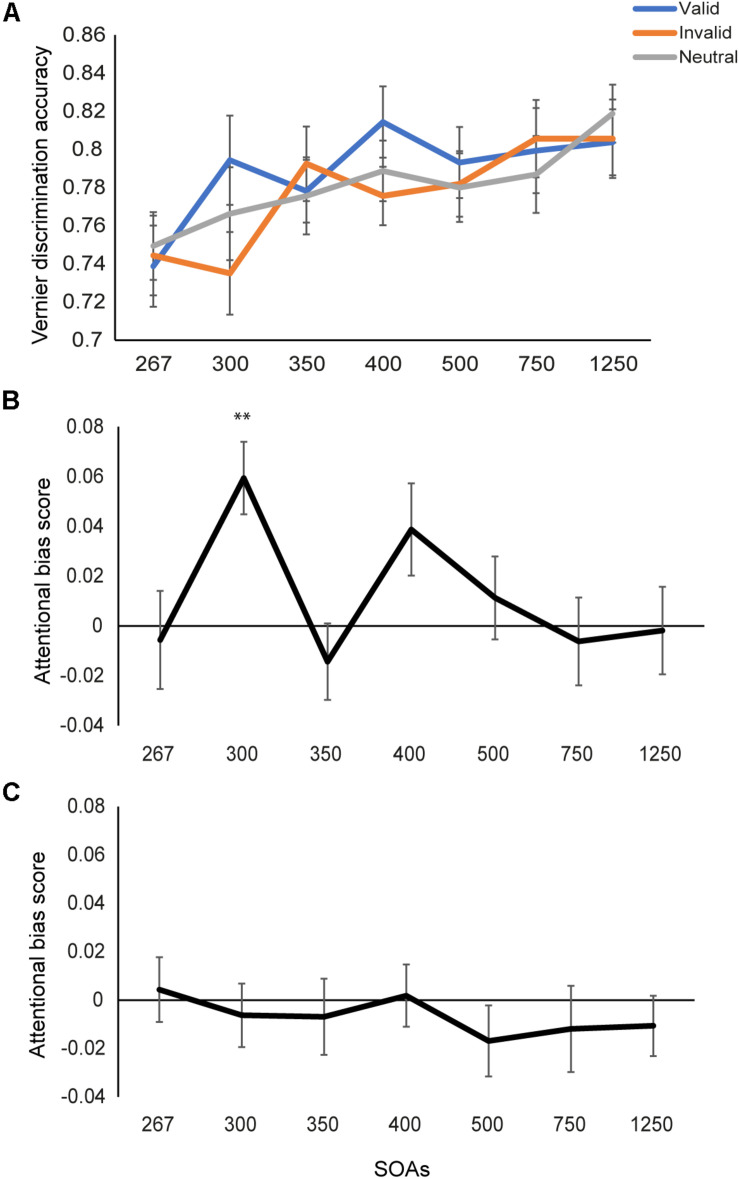
Results of Experiments 1 and 2. **(A)** Discrimination accuracy for the Vernier task under different cueing conditions as a function of SOA in Experiment 1. Vernier discrimination performance increased as SOA increased. **(B)** The emotional attention effect by comparing accuracy in the valid cue with that in the invalid cue condition. The significant attentional effect was found at the SOA of 300 ms. **(C)** The emotional attention effect in Experiment 2. No attentional cueing effect when the face cues were presented inverted. ^∗∗^*p* < 0.01, Bonferroni corrected. Error bars represent standard error of the mean.

Percentage of accuracy for Vernier discrimination was calculated. We performed a 3 (cue types: valid, invalid, and neutral) × 7 (SOAs) repeated measures ANOVA on the accuracy. A significant main effect was found for SOAs (*F*(6,114) = 9.97, *p* < 0.001, η^2^ = 0.34), with a larger SOA being accompanied with better performance on the Vernier task (Figure 2A). The main effect of cue type was marginally significant (*F*(2,38) = 2.87, *p* = 0.069, η^2^ = 0.13). Further inspections found higher accuracy for the valid- than invalid-cue condition (*p* = 0.023), demonstrating the attentional effect of fearful faces. The interaction between the three cue types and seven SOAs were not significant (*F*(12,228) = 1.58, *p* = 0.10, η^2^ = 0.08).

Next, we tested the emotional attention effect directly by comparing the valid- and invalid-cue conditions. An attentional bias score (Accuracy_valid_ - Accuracy_invalid_) was calculated for each SOA (Figure 2B). To examine the dynamics of the emotional attention effect, we performed one-way ANOVA on the attentional bias score across the 7 SOAs. A significant effect (*F*(6,114) = 2.33, *p* = 0.04, η^2^ = 0.11) was found, demonstrating that the emotional attention effect varied across the 7 SOAs. Simple effect analysis (comparing the attentional bias scores between each two SOAs) found larger attentional bias at the SOA of 300 ms than the remaining SOAs (*t*s > 2.11, *p*s < 0.05, uncorrected) excepting the SOA of 400 ms (*t*(19) = 1.13, *p* = 0.27). No difference was found between other SOAs (ts < 2.0, *p*s > 0.06).

The above findings were further confirmed by examining the emotional attention effect (Accuracy_valid_ versus Accuracy_invalid_) at each SOA. A significant attentional effect was found at the SOA of 300 ms (*t*(19) = 4.08, *p* < 0.01, Bonferroni corrected), namely facilitation of visual acuity occurred when the target was presented 50 ms after the attentional cue. No significant attentional effect was found when the SOA was shorter or longer (*p*s > 0.05 for the remaining SOAs).

## Experiment 2

The results in Experiment 1 suggested an attentional effect of fearful faces on visual acuity at a latency of around 300 ms. However, before concluding the effect results from emotion, we need to exclude the possible contribution of physical differences between the fearful and neutral expressions. The face cues were presented inverted as to eliminate the emotional effect but hold/keep/maintain the physical feature similar with the upright face. To keep the physical differences while eliminating the emotional effect (McKelvie, 1995), the face cues were presented inverted. The same Posner attention test was given.

### Methods

#### Participants

The same 20 participants were recruited again for Experiment 2. All of them conducted Experiments 1 and 2 on separate days.

#### Stimuli and Procedure

The same stimuli were used in Experiment 2 as in Experiment 1. The procedure was the same except that all the face images were presented upside down.

### Results

A 3 (cue types: valid, invalid, and neutral) × 7 (SOAs) repeated measures ANOVA was performed on the Vernier discrimination accuracy. A significant effect of SOAs was found (*F*(6,114) = 13.61, *p* < 0.001, η^2^ = 0.42), with higher accuracy for longer SOA. No significant effect of cue types was found any more (*F*(2,38) = 1.85, *p* = 0.17, η^2^ = 0.09). No interaction effect between the two factors was found either (*F*(12,228) = 0.46, *p* = 0.94, η^2^ = 0.02). We then tested the attentional effect (i.e., valid vs. invalid cue condition) at each SOA (Figure 2C). Different from Experiment 1, when faces were presented upside down, no attentional effect was found (all ts < 1.20, *p*s > 0.05) under any SOA. Therefore, the emotional cueing effect was not caused by physical differences between the fearful and neutral faces when the faces were presented inverted.

## Experiment 3

Findings in Experiments 1 and 2 provide evidence that fearful faces attract attention and facilitate visual acuity at its location. To confirm the above findings, we performed Experiment 3 using the same procedure. A few changes were made. First, faces were presented for 150 ms, shorter than in Experiment 1 but long enough time for emotion processing. Second, SOAs were restrained to 183 – 450 ms in order to refine the time course of the emotional attention effect. In this way, we could explore whether the duration of the emotional cue or the SOA between the cues and targets is more important in determining the emotional attention effect.

### Methods

#### Participants

Thirteen naïve participants (7 females) with normal or corrected to normal vision were recruited. They were right-handed and had no known neurological or visual disorders. They gave written, informed consent in accordance with procedures and protocols approved by the human subject review committee of Peking University.

#### Stimuli and Procedure

The same stimuli were used as in Experiment 1. The procedure was the same as Experiment 1 with two major differences. First, all the face images were presented for 150 ms, followed by a blank interval of 33, 50, 100, 150, 200, 250, or 300 ms. Therefore, the SOAs were 183, 200, 250, 300, 350, 400, and 450 ms. Second, the Vernier task was changed to discriminate the misalignment of two vertical segments. At the same time, two horizontal segments with no offset were presented at the opposite side of the vertical target as a distractor. Participants were instructed to discriminate whether the top segment laid left or right to the bottom segment in the target while ignoring the distractor.

### Results

A 3 (cue types: valid, invalid, and neutral) × 7 (SOAs) repeated measures ANOVA was performed on the Vernier discrimination accuracy. A significant interaction effect between the cue types and SOAs was found (*F*(12,144) = 1.80, *p* = 0.05, η^2^ = 0.13), indicating the attentional cueing effect varied at different SOAs. Significant main effect of SOAs (*F*(6,72) = 4.24, *p* = 0.001, η^2^ = 0.26) but not cue types (*F*(2,24) = 1.42, *p* = 0.26, η^2^ = 0.11) was found.

We calculated the attentional bias score (Accuracy_valid_ - Accuracy_invalid_) at each SOA and compared the attentional bias scores across different SOAs. One-way ANOVA on the attentional bias score across the 7 SOAs found marginal significant effect (*F*(6,72) = 1.99, *p* < 0.08, η^2^ = 0.14). Simple effect analysis (comparing the attentional bias scores between each two SOAs) found larger attentional bias at the SOA of 300 ms than the remaining SOAs (*t*s > 2.48, *p*s < 0.03, uncorrected) excepting the SOA of 250 ms (*t*(12) = 1.39, *p* = 0.19). No difference was found between any other SOAs (*t*s < 1.0, *p*s > 0.33).

**FIGURE 3:**
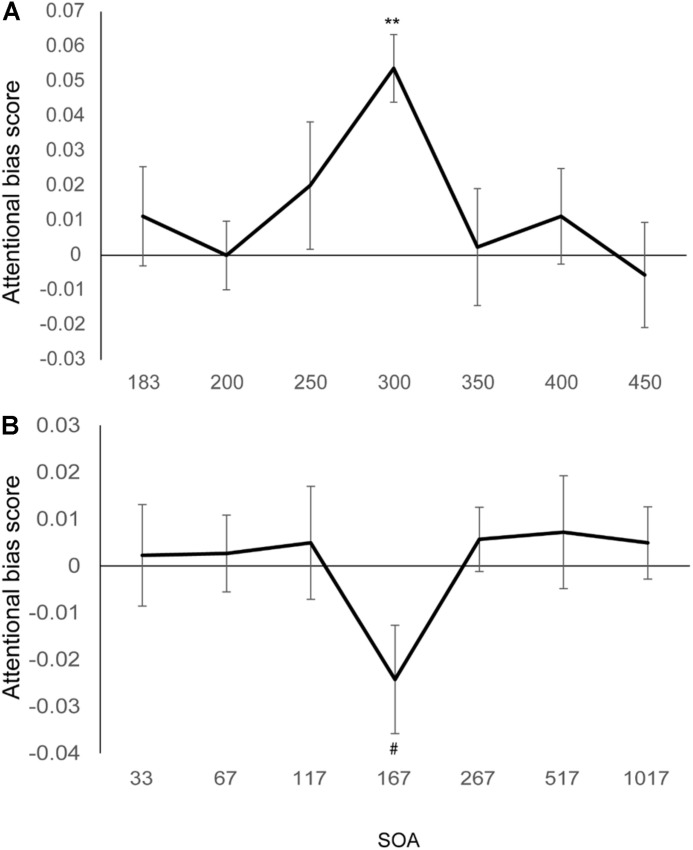
Results of Experiments 3 and 4. **(A)** The emotional attention effect of Experiment 3 by comparing accuracy in the valid cue with that in the invalid cue condition. The improvement was found at the SOA of 300 ms. **(B)** The emotional attention effect of Experiment 4. No improvement but a trend of interference was found at the SOA of 167 ms. **p < 0.01, Bonferroni corrected; #*p* < 0.05, uncorrected. Error bars represent standard error of the mean.

We then tested whether the attentional effect was significant at each SOA. The same perceptual facilitation effect was found (see Figure 3A, *t*(12) = 5.53, *p* < 0.01, Bonferroni corrected) at the same SOA of 300 ms as in Experiment 1, though the cue duration was shortened. The cueing effect was not significant in other SOAs (all *t*s < 1.1, all *p*s > 0.29). Therefore, fearful faces attract attention and facilitate visual acuity. The facilitation happens around 300 ms SOA (from stimulus onset).

## Experiment 4

The consistent findings that emotional attention improved visual acuity in Experiments 1 and 3 comply with the priority of emotional information in attracting attention (Ohman and Mineka, 2001; Pessoa and Adolphs, 2010). However, would perceptual facilitation still happen when processing emotional information already occupy the attentional resource? To answer this question, we increased the difficulty of emotion processing by presenting the face cues very briefly in Experiment 4. The face cues were presented for 17 ms, without backward or forward masking. Thus, participants would always perceive the faces. A Vernier discrimination task was given after 17, 50, 100, 150, 250, 500, or 1000 ms. The attentional effect was tested again for the minimally perceived fearful faces. We hypothesize that threatening faces couldn’t facilitate perceptual discrimination when the presentation of emotional faces in a very short time (17 ms). When the threatening information of the emotional faces was presented as short as 17 ms, attention would be used preferentially for emotion processing. This might cause difficulty in disengaging attention from the emotional faces and thus disrupt subsequent perceptual discrimination.

### Methods

#### Participants

Twenty-two naïve participants (10 females) with normal or corrected to normal vision were recruited. They were right-handed and had no known neurological or visual disorders. They gave written, informed consent in accordance with procedures and protocols approved by the human subject review committee of Peking University.

#### Stimuli and Procedure

The same stimuli were used as in Experiment 1. The procedure was the same except that (1) all the face cues were presented for 17 ms and (2) only valid- and invalid-cue conditions were retained. Note that the interval between the face images and the probe was not changed. Thus, the shorter cue presentation resulted in shorter SOAs, which were 34, 67, 117, 167, 267, 517, and 1017 ms.

### Results

A 2 (cue types: valid and invalid) × 7 (SOAs) repeated measures ANOVA was performed on the accuracy of the probe task. Only a significant main effect of SOAs was found (*F*(6,126) = 31.89, *p* < 0.001, η^2^ = 0.60). Neither the main effect of cue types (*F*(1,21) = 0.02, *p* = 0.89, η^2^ = 0.001) or the interaction effect of the two factors (*F*(6,126) = 1.16, *p* = 0.33, η^2^ = 0.05) was significant.

We further calculated the attentional bias score (see Figure 3B, Accuracy_valid_ - Accuracy_invalid_) of each SOA. One-way ANOVA on the attentional bias score across the 7 SOAs found no significant effect anymore (*F*(6,126) = 1.16, *p* = 0.33, η^2^ = 0.05). No attentional facilitation effect (valid vs. invalid cue condition) was found when under any SOAs either. In contrast, a trend of attentional cost was detected at an SOA of 167 ms (*t*(21) = −2.09, *p* = 0.049, uncorrected). Therefore, the level of processing on the fearful emotion could possibly affect emotional attention. When the fearful face was presented abruptly and in a very short duration (17 ms), it tended to induce attentional deficits and damage visual acuity. However, this finding should be confirmed in further studies.

## Results of Reaction Time for Experiment 1, 2, 3, and 4

For the Vernier task in all four experiments, accuracy is important and effective in indicating the performance. Nevertheless, we also analyzed the attentional effect on reaction time (RT). Repeated measures ANOVAs were performed on the RT results for all four experiments. However, no significant interaction effect was found for any experiment. No attentional effect was found overall or under each SOA (*p*s > 0.05). The main effects of SOA were significant for Experiments 1, 2, and 4, indicating that RT reduced with SOA. No other significant main effects were found. These results indicate that there was no RT-accuracy trade-off in the present study.

## Discussion

Previous evidence about the emotion-attention interactions on basic perception showed that emotion can potentiate the effect of spatial attention (Bocanegra and Zeelenberg, 2011a). The current study further demonstrated that fearful emotion improved visual acuity, as evidenced by higher accuracy in Vernier discrimination at the same than the opposite location of a preceding fearful face cue. However, observation of the improvement effect is not unconditional. First, when a series of SOAs were examined, an improvement on visual acuity was detected consistently at an SOA of around 300 ms, suggesting that emotional attention facilitation was most evident at a specific period after stimulus onset. Moreover, emotion’s effect on visual acuity heavily relied on its interactions with attention. On the one hand, when a fearful face cue was presented long enough (i.e., Experiments 1 and 3: ≥ 150 ms), the threatening emotion attracted attention and improved visual acuity at its location. On the other hand, when a fearful face cue was presented abruptly (Experiment 4: 17 ms), a weak cost was found on the cued location, which indicates that the very short stimulus might occupy the attentional resource and cause difficulty in disengaging attention from the face. Our findings suggest that the fearful emotion is presented more than 150 ms which is long enough for people to perceive and process the threaten information to potentiate the effects of attention.

Facilitation of emotion on low-level visual perception has been reported in previous studies. Inspired by the neuroimaging findings that emotional stimuli enhanced neural activity in early visual cortical areas (Padmala and Pessoa, 2008), Phelps et al. (2006) examined contrast sensitivity, an early visual process, on Gabor patches after cueing by a fearful or neutral face. In their study, participants showed heightened contrast sensitivity after a fearful face and this effect was modulated strongly by how much attention could be given to the target location. This result provided the first piece of behavioral evidence for emotional stimuli on early visual processes. Several researches (Bocanegra and Zeelenberg, 2009; Bocanegra and Zeelenberg, 2011a; Lee et al., 2014; Song and Keil, 2014) further verified that this improvement was restricted to low-spatial-frequency information, corresponding to the function of Amygdala in low-spatial-frequency visual information processing. The current study tested how the fearful emotion affected visual acuity. Two experiments (Experiments. 1 and 3) consistently showed an improvement on visual acuity after showing a fearful face, and the effect could not be attributed to low-level feature differences because it did not exist anymore when the same faces were presented upside down (Experiment. 2). Therefore, the effect of emotional attention on visual perception is likely to be general.

The current study and previous studies consistently demonstrated that fearful faces, and other threat- relevant information, may automatically affect our attention (Fox et al., 2001, 2002; Ohman et al., 2001; Armony and Dolan, 2002; Koster et al., 2004; Pourtois et al., 2004; Cooper and Langton, 2006; Salemink et al., 2007; Carlson et al., 2009). Specifically, our results provided further evidence to the time course of the emotional attentional effect on visual acuity. In the field of emotion and attention, durations of between 100 and 500 ms post-stimulus onset are thought necessary for attentional processing to become active by using threat signals (Koster et al., 2005; Cooper and Langton, 2006; Mogg and Bradley, 2006). For example, a study examining the time-course for attentional bias found that fearful faces captured and held attention at short durations (SOAs) within and approximate to 300 ms (Torrence et al., 2017). Our results extended previous findings by showing an attentional effect induced by fearful emotion at a short SOA of 300 ms. We varied the SOA so that the visual acuity test was given at several latencies after the processing of the emotional or unemotional faces. Interestingly, the improvement on visual acuity has been consistently found at an SOA of 300 ms, when the faces were processed for 150 ms (Experiment 3) or 250 ms (Experiment 1). Therefore, attention was attracted to the location of fully-processed fearful faces around 300 ms after the faces were presented.

Previous researches examined the time course of emotional attention in the normal population showed an active window from 100 to 1500 ms. Several dot-probe studies have observed the time-course of attention bias for threatening stimuli faces with relatively short SOAs (∼100 ms) in normal samples (Koster et al., 2005; Cooper and Langton, 2006). Phelps et al. (2006) and Bocanegra and Zeelenberg (2009, 2011b) detected perceptual improvement 115 and 100 ms after cue onset. The current study did not test such short SOAs. However, depending on the fact that the effect did not exist between 183 ms (150 ms + 33 ms, Experiment 3) and 267 ms (250 ms + 17 ms, Experiment 1), we tend to believe that the attentional effect in our manipulation is a relatively slower process. Notably, the effect of emotional attention is not necessarily fast. The time course depends on the task. For example, using cues that were conditioned to be fearful, contrast sensitivity improvement happened 1700 ms after cue onset (Song and Keil, 2014). The slower effect in the current study may thus be due to the Vernier discrimination task being used. Future studies could test whether the same time course persists for other measurements of visual acuity. The time course of emotional attention has also been studied by EEG and MEG. Amplified responses to emotional visual events have been found in early (e.g., P1 and N1 at 120–150 ms), middle (e.g., N2, 200–300 ms), and late (e.g., P3, 300–400 ms) cognitive components. A review (Olofsson et al., 2008) summarized the effects of different emotional components on ERP responses and suggested that valence exerts influence on early and middle components and arousal primarily affects the middle-range and later ERP components. Therefore, valence and arousal may both be effective between 200 and 400 ms, which was consistent with our results. Nevertheless, further studies are needed to get a more complete picture of the time course of the emotional attention effect.

We had 20, 20, and 22 subjects in Experiment 1, Experiment 2, and Experiment 4, respectively. In these experiments, the sample size of 20 subjects had sufficient power to get significant results. For example, for the significant attentional bias effect of 300 ms, it was found that with effect size = 1 and power = 0.95, only 13 subjects could result in a significance. Experiment 3 was designed to validate the findings in Experiment 1. As the significant effect had been found in Experiment 1, Experiment 3 did not need to recruit the same sample size (*n* = 20) as in Experiment 1. Therefore, we had 13 participants in Experiment 3, and didn’t recruit more.

In addition to facilitation, the current study detected a weak interference effect of emotional attention on visual acuity. This happens when the fearful and neutral faces were presented at a very short duration (i.e., 17 ms, Experiment 4). The duration of 17 ms has been used to study unconscious emotion processing when combined with a neutral mask (Pessoa and Adolphs, 2010). It could be inferred from our results that the presentation might be too short to provide sufficient information for perceptual and emotional processing. And if a backward mask is given, it prevents the visual system from processing the limited visual information and thus resulted in unconsciousness of the emotional stimulus. However, when without the mask, people are well aware of the existence of an emotional stimulus. As reported by participants in Experiment 4, the 17 ms duration is too short to process the emotion fully in the faces. This abruptly fearful face might attract too much attention and cause difficulty in disengaging attention from preparing for the ambiguous danger. This result resembles a finding (Koster et al., 2007) of delayed responding on trials with briefly presented emotional cues, indicating that briefly presenting faces might occupy more cognitive resources. The lack of facilitation and the trend of interference on visual acuity by the briefly presented fearful face cues also indicates that some degree of attention is necessary for emotion processing to occur (Pessoa et al., 2002). However, two facts should be noticed. First, Experiment 4 kept the inter-stimulus interval (ISI, interval between the disappearance of cue and the appearance of probe) but not the SOA the same as in Experiment 1. As a result, Experiment 4 lacked a condition of 300 ms SOA. This is one limitation in our study, making it hard to compare the results from Experiment 1 and Experiment 4 directly. Second, the finding of attentional cost was relatively weak, and could not pass correction of multiple comparisons. Therefore, the result from Experiment 4 only showed a trend on the attentional effect of temporarily processed fearful face. Further studies are needed to confirm this finding.

To summarize, threatening emotion not only enhances the processing of its own but also facilitates low-level perceptual processes. We found that fearful emotion may affect visual processing through attentional modulation, which is consistent to previous studies (Bocanegra and Zeelenberg, 2011a; Ferneyhough et al., 2013; Barbot and Carrasco, 2018). Most importantly, the present results demonstrate the time course of the attentional modulation induced by fearful faces, which is elusive in previous studies. Specifically, we not only demonstrate a restriction of processing time for emotional cues to facilitate visual acuity, but also detect a dynamic interaction between emotion and attention. Future studies testing the emotional attention effect should pay careful attention to the presentation time of the emotional cues, too long or too short duration might produce different effects. Furthermore, future studies can delve deeper into the influence of duration and awareness of the emotional cue on attention as well as perceptual processing. The oscillation of the emotional attention effect, although is inconclusive, suggests further investigations using more sensitive approaches.

## Data Availability Statement

The raw data supporting the conclusions of this article will be made available by the authors, without undue reservation.

## Ethics Statement

The studies involving human participants were reviewed and approved by School of Psychological and Cognitive Sciences, Peking University, China. The patients/participants provided their written informed consent to participate in this study.

## Author Contributions

TB designed the study. ZS conducted the study. TB and ZS did the data analysis. YW, ZS, and TB wrote the manuscript. All authors contributed to the article and approved the submitted version.

## Conflict of Interest

The authors declare that the research was conducted in the absence of any commercial or financial relationships that could be construed as a potential conflict of interest.
